# Function and evolution of the serotonin-synthetic *bas-1 *gene and other aromatic amino acid decarboxylase genes in *Caenorhabditis*

**DOI:** 10.1186/1471-2148-4-24

**Published:** 2004-08-02

**Authors:** Emily E Hare, Curtis M Loer

**Affiliations:** 1 Department of Biology, University of San Diego, 5998 Alcala Park, San Diego, CA 92110, USA; 2 current address: Department of Molecular and Cell Biology, University of California, Berkeley, CA 94720, USA

## Abstract

**Background:**

Aromatic L-amino acid decarboxylase (AADC) enzymes catalyze the synthesis of biogenic amines, including the neurotransmitters serotonin and dopamine, throughout the animal kingdom. These neurotransmitters typically perform important functions in both the nervous system and other tissues, as illustrated by the debilitating conditions that arise from their deficiency. Studying the regulation and evolution of AADC genes is therefore desirable to further our understanding of how nervous systems function and evolve.

**Results:**

In the nematode *C. elegans*, the *bas-1 *gene is required for both serotonin and dopamine synthesis, and maps genetically near two AADC-homologous sequences. We show by transformation rescue and sequencing of mutant alleles that *bas-1 *encodes an AADC enzyme. Expression of a reporter construct in transgenics suggests that the *bas-1 *gene is expressed, as expected, in identified serotonergic and dopaminergic neurons. The *bas-1 *gene is one of six AADC-like sequences in the *C. elegans *genome, including a duplicate that is immediately downstream of the *bas-1 *gene. Some of the six AADC genes are quite similar to known serotonin- and dopamine-synthetic AADC's from other organisms whereas others are divergent, suggesting previously unidentified functions. In comparing the AADC genes of *C. elegans *with those of the congeneric *C. briggsae*, we find only four orthologous AADC genes in *C. briggsae*. Two *C. elegans *AADC genes – those most similar to *bas-1 *– are missing from *C. briggsae*. Phylogenetic analysis indicates that one or both of these *bas-1*-like genes were present in the common ancestor of *C. elegans *and *C. briggsae*, and were retained in the *C. elegans *line, but lost in the *C. briggsae *line. Further analysis of the two *bas-1*-like genes in *C. elegans *suggests that they are unlikely to encode functional enzymes, and may be expressed pseudogenes.

**Conclusions:**

The *bas-1 *gene of *C. elegans *encodes a serotonin- and dopamine-synthetic AADC enzyme. Two *C. elegans *AADC-homologous genes that are closely related to *bas-1 *are missing from the congeneric *C. briggsae*; one or more these genes was present in the common ancestor of *C. elegans *and *C. briggsae*. Despite their persistence in *C. elegans*, evidence suggests the *bas-1*-like genes do not encode functional AADC proteins. The presence of the genes in *C. elegans *raises questions about how many 'predicted genes' in sequenced genomes are functional, and how duplicate genes are retained or lost during evolution. This is another example of unexpected retention of duplicate genes in eukaryotic genomes.

## Background

**Figure 1:**
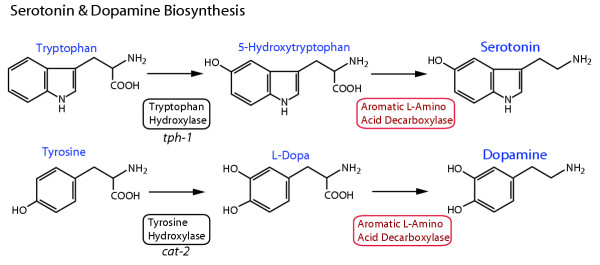
**Serotonin and dopamine biosynthetic pathways. **Serotonin and dopamine are synthesized from the aromatic amino acids tryptophan and tyrosine, respectively. The first and rate-limiting step in synthesis is carried out by a neurotransmitter-specific aromatic amino acid hydroxylase enzyme, either tryptophan or tyrosine hydroxylase. In *C. elegans*, these genes are encoded by the *tph-1 *and *cat-2 *genes, respectively [19, 25]. The second synthetic step for both neurotransmitters shares the aromatic L-amino acid decarboxylase (AADC) enzyme, which has a relatively broad substrate specificity, and is also known as 5-hydroxytryptophan decarboxylase or dopa decarboxylase.

Aromatic L-amino acid decarboxylase (E.C. 4.1.1.28, AADC) catalyzes the second enzymatic step in synthesis of the neurotransmitters dopamine and serotonin, which are found in neurons of all animals (Figure 1). Alteration in the normal expression of these transmitters is associated with human neurological disorders such as Parkinson's disease and depression [1,2]. In mammals, AADC is expressed in many tissues beside the nervous system, associated with additional regulatory roles of dopamine and serotonin in a wide range of tissues [3]. In insects, AADC is further required to produce amines for cuticle synthesis and pigmentation [4]. Because of its role in the synthesis of both transmitters, by decarboxylation of L-dopa and 5-hydroxytryptophan, AADC is also known as dopa decarboxylase or 5-hydroxytryptophan decarboxylase (reviewed in [3]). AADC belongs to the α family (subgroup II) of pyridoxal-5'-phosphate (PLP) dependent enzymes. Other subgroup II enzymes include histidine, tyrosine, tryptophan and glutamate decarboxylases [5]; in animals some of these enzymes mediate synthesis of other biogenic amines (e.g., histamine, tyramine, octopamine) and GABA. In mammals and in *Drosophila*, a single gene encodes the serotonin- and dopamine-synthetic AADC [6,7], although tissue-specific isoforms of the protein are generated by alternative splicing [8,9]. Different genes encode PLP-dependent decarboxylase enzymes for histamine, octopamine and GABA synthesis.

In the nematode *Caenorhabditis elegans*, serotonin is expressed in at least nine neurons in the hermaphrodite and nineteen in the male; dopamine is found in eight neurons in the hermaphrodite and fourteen in the male [10]. By examining the behavior of worms in which specific neurons have been ablated and examining mutants lacking serotonin and/or dopamine, we have learned that serotonin is involved in behaviors including egg laying [11-13], pharyngeal pumping [14,15], male mating [16], and experience-dependent regulation of locomotion [17,18]. Serotonin-deficient mutants also display abnormalities in entry into the diapause-like dauer stage and in fat storage, mediated via an insulin-related signaling pathway [19,20]. Dopamine plays roles in male mating [21], in regulating locomotion via mechanosensation [17,22], and in foraging behavior [23].

Identification of genes involved in neurotransmitter synthesis and related aspects of signaling in *C. elegans *was greatly accelerated by genomic sequencing, which was essentially completed in 1998 [23,24]. For genes identified originally by mutants via a traditional genetic approach, a candidate gene approach often allowed rapid confirmation of a gene's identity; for predicted genes identified from the genomic sequence by homology, a reverse genetic approach has been taken. Many components of the serotonin and dopamine synthesis and transport pathways in *C. elegans *have now been identified by these traditional and reverse genetic approaches, including tyrosine hydroxylase (*cat-2*; [25]), tryptophan hydroxylase (*tph-1*; [19]), serotonin reuptake transporter (*mod-5*; [26]), dopamine reuptake transporter (*dat-1*; [27]) and vesicular monoamine transporter (*cat-1*; [28]). Postsynaptic components have also been identified, including various receptors [29-32] and intracellular G protein signaling components [33-36].

Further analysis of gene function, regulation and evolution in *C. elegans *is being facilitated by genomic sequencing of related nematodes. A whole genome shotgun sequence of *Caenorhabditis briggsae *was recently completed; the sequence is estimated to be 98% complete [37]. The divergence of *C. briggsae *and *C. elegans *is estimated between 80 – 110 million years ago [37,38], although it should be noted that these estimates lack a fossil record to anchor the dates [39]. This is considered to be a favorable evolutionary distance to identify conserved non-coding regulatory sequences, although the sequences from only two orthologous genes from related species is often inadequate to identify such sequences unambiguously. Genomic sequencing is planned or underway of three additional congeneric relatives of *C. elegans *that are more closely related than *C. briggsae*, which will enhance our ability to analyze the genes of *C. elegans*. We have used genomic sequences of both *C. elegans *and *C. briggsae *to help identify and characterize another component of the serotonin and dopamine signaling systems – the *bas-1 *gene – and to examine the evolution of this and related genes.

The *bas-1 *[*b*iogenic *a*mine *s*ynthesis abnormal] mutant is serotonin- and dopamine-deficient, and displays several behavioral abnormalities [12,16,17]. Unlike wildtype and other serotonin-deficient mutant worms, *bas-1 *mutants are unable to convert exogenous 5-hydroxytryptophan (5HTP) into serotonin (5-hydroxytryptamine, 5HT), as assessed by serotonin antiserum staining. Because of this phenotype, we have previously proposed that the *bas-1 *gene likely encoded the AADC enzyme of *C. elegans *[16].

## Results

### Rescue of the *bas-1 *mutant with an AADC-homologous sequence

**Figure 2:**
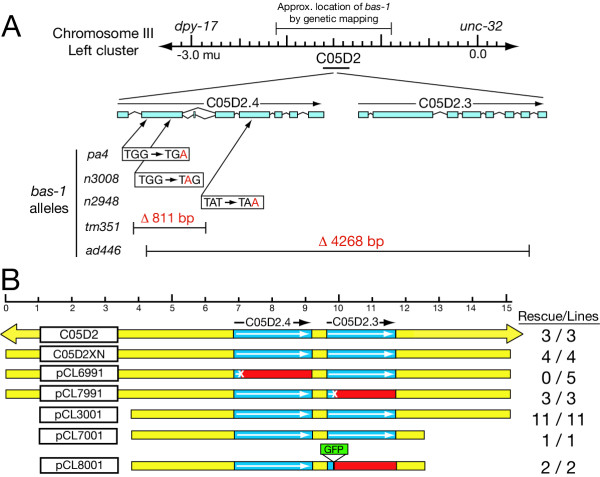
**Molecular genetics and transformation rescue of *bas-1. ***(A) Genetic and physical map of *bas-1 *region and *bas-1 *mutant alleles. Locations and extent of mutations for each *bas-1 *allele are shown to scale with respect to C05D2.4 and C05D2.3 coding sequences, based on known splicing patterns or Genefinder predictions. Exons are indicated by rectangular bars; an alternatively spliced 27 bp exon is also indicated after exon 2 in C05D2.4. Four of five *bas-1 *mutants affect only C05D2.4; *ad446 *is a larger deletion removing most coding sequence of both C05D2.4 and C05D2.3. (B) Genomic DNA constructs that rescue or fail to rescue *bas-1 *mutants. Constructs are shown to scale (top), based on the 15.1 kbp insert of the plasmid clone C05D2XN; construct names are indicated in the box on the left. The cosmid C05D2 is larger, as indicated by the arrows. Clones below are subclones or modifications of C05D2XN. Coding regions for the two predicted AADC genes C05D2.4 and C05D2.3 are indicated by the blue boxes; intergenic regions are shown in yellow. C05D2XN upstream of C05D2.4 contains two other predicted genes, one complete (C05D2.8) and one partial (C05D2.5). There are no predicted genes in the 3 kb downstream of C05D2.3. In the constructs with the least upstream sequence, only a portion of C05D2.8 remains. Constructs mutated to introduce premature stop codons are indicated with a X in the coding sequence, and red downstream of the introduced stop codon. The construct pCL8001 has a GFP gene inserted in a manner that would inactivate the C05D2.3 gene, so is comparable to the pCL7991 construct. [No GFP expression was seen in the C05D2.3::GFP reporter construct lines.]

The *bas-1 *gene maps to chromosome III, between *dpy-17 *and *unc-32*. When this region was sequenced by the *C. elegans *Genome Sequencing Consortium, two AADC-homologous predicted genes, designated C05D2.4 and C05D2.3, were found to be located close together on a single cosmid, C05D2 (Fig. 2A). This suggested that one (or both) of these sequences comprised the gene mutated in *bas-1 *mutants. To test this hypothesis, we injected *bas-1 *mutants with the cosmid C05D2 plus *rol-6 (dom) *plasmid DNA as a co-injection marker. We isolated transgenic Roller progeny (expressing the *rol-6 (dom) *marker phenotype) of the injected worm and propagated strains that transmitted the marker, then tested these worm strains using serotonin antibody staining. We found that 3 of 3 independent Roller transgenic lines were rescued for serotonin immunoreactivity, confirming that the *bas-1 *gene was located within this 46 kb of genomic DNA (Fig. 2B). We then injected plasmid subclones of C05D2, each of which still contained both the predicted C05D2.4 and C05D2.3 genes. A 15.1 kb plasmid subclone (C05D2XN) also rescued *bas-1 *mutants (n = 4/4), as did smaller subclones of C05D2XN, including an 11.3 kb subclone (pCL3001, n = 11/11) and an 8.8 kb subclone (pCL7001, n = 1/1). These results confirm that at least one of AADC-homologous genes likely corresponds to the *bas-1 *gene.

To determine which of the two predicted AADC sequences was needed to rescue *bas-1 *mutants, we prepared two constructs from C05D2XN, one mutated in C05D2.4, the other in C05D2.3 (Fig. 2B). In each case, a mutation was created by eliminating a unique restriction site early in the predicted coding region, creating a frameshift resulting in premature stop codons. We found that constructs mutated in C05D2.3 when injected rescued serotonin immunoreactivity in *bas-1 *mutants (n = 3/3), whereas the construct mutated in C05D2.4 failed to rescue (n = 0/5 rescued). A construct containing a GFP gene inserted into the C05D2.3 coding sequence (and disrupting the gene) also rescued *bas-1 *mutants (n = 2/2). In Roller transgenic lines lacking rescue, we confirmed the presence of the injected construct by PCR. Therefore, an intact C05D2.4 gene is necessary to rescue *bas-1 *mutants, whereas the C05D2.3 gene is not. In all rescued transgenic lines, we saw the complete set of known serotonergic neurons, although not necessarily all cells in every animal – mosaicism from somatic loss of extrachromosomal DNA is expected in these transgenics. This result suggests that no critical cell-specific regulatory sequences were missing from even the smallest construct we injected.

To confirm further that C05D2.4 is the *bas-1 *gene, we identified the mutations in four *bas-1 *mutant alleles; we also examined the phenotypes of deletion mutants in C05D2.4 and C05D2.3 generated by the *C. elegans *Gene Knockout Consortium (GKC). We found that the *bas-1 *alleles *pa4*, *n2948*, and *n3008 *contained point mutations in C05D2.4 coding sequence resulting in premature stop codons (Fig. 2A). We found that the original *bas-1 *allele (*ad446*) had a 4268 bp deletion from the second exon of C05D2.4 to the final intron of C05D2.3; therefore, *ad446 *is a knockout of both predicted genes. We examined the phenotypes of GKC-generated deletion mutants in each predicted gene. The C05D2.4 knockout (*tm351*) removes the entire predicted second exon. We found that both *tm351 *homozygotes and *tm351/ad446 *worms were deficient in serotonin immunoreactivity. On the other hand, a knockout of C05D2.3 (*ok703*) is wildtype for serotonin staining. Therefore, *tm351 *is a fifth mutant allele of the *bas-1 *gene, and C05D2.4 corresponds to the gene *bas-1*.

### Transcripts from the *bas-1 *gene and the predicted BAS-1 protein

**Figure 3:**
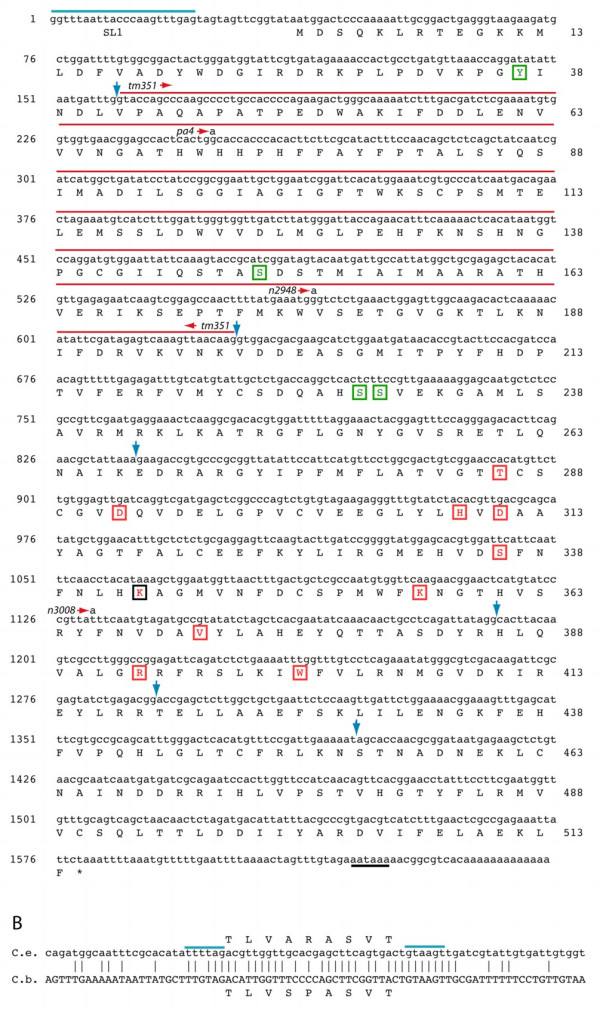
***C. elegans bas-1 *cDNA sequences. **(A) Consensus cDNA sequence and translation for C05D2.4/*bas-1*, based on the most common splice form. Nucleotide numbering is shown on the left side and amino acid numbering on the right side of the sequence. SL1 spliced leader sequence is overlined in blue in the top line. Intron locations are indicated with blue arrowheads; the phase at all intron locations is 0 (between codons; see also Fig 6). The conserved lysine (K) pyridoxal 5-phosphate binding site at amino acid 343 is boxed in black. Red amino acids in the predicted Bas-1 protein (T286, D292, H309, D311, S336, K343, K357, V378, R393, and W401) are identical to those shown to be essential for rat AADC function [5, 41, 42]. Possible phosphorylation sites that are absolutely conserved in known DDC and HisDC proteins are boxed in green (Y37, S149, S229, S230). The polyadenylation signal in the final line is underlined. Mutations found in bas-1 alleles are indicated with the allele designation and the changed base over the wildtype sequence. The allele tm351 deletion, which removes the entire second exon, is indicated by a red line over the missing sequence. The wildtype cDNA sequence shown is consistent with our RT-PCR clones (primers SL1B, C05D2-B), those we sequenced from the ORFeome project (from predicted translation start to stop), and *C. elegans *EST project 'YK clones' ends (used to determine the 3' end, including the site of polyadenylation). (B) Alternatively spliced 27 bp exon and surrounding genomic sequence in *C. elegans *and *C. briggsae*. The additional exon is found in a fraction of *C. elegans bas-1 *transcripts, and the sequence is conserved in genomic sequence from *C. briggsae *as shown. [We did not isolate a cDNA containing this exon among our *C. briggsae bas-1 *cDNAs, S. DePaul & C. Loer, unpublished results.] Predicted translation of the exon is shown above or below the nucleotide sequence. Consensus splice signals are overlined in blue, and identical nucleotides are indicated by vertical lines between the two nucleotide sequences.

To continue our characterization of the *bas-1 *gene, we isolated cDNAs using RT-PCR; we also obtained cDNA clones from the *C. elegans *EST/Transcriptome project (courtesy of Yuki Kohara) and the ORFeome project [40]. We found that C05D2.4/*bas-1 *cDNAs are trans-spliced to SL1 just 15 nucleotides upstream of the predicted translation start site. The consensus sequence from our clones and others we examined predicts a 514 amino acid, 58 kDa protein product (Fig. 3A). This is similar in size to other known AADC/dopa decarboxylase proteins such as those of *Drosophila *(510 aa) and human (480 aa). The predicted protein possesses a conserved lysine PLP binding site at residue 343, and has other amino acids identical to those shown to be essential for rat AADC function [5,41,42]. A number of possible phosphorylation sites can be predicted, including three serines and one tyrosine that are conserved in all known AADCs and HisDCs (Fig. 3A).

We found two splice variants different from the Genefinder-predicted cDNA described above, which was the predominant form. About 20% of clones we sequenced had a 27 bp microexon inserted between the predicted exons 2 and 3 (Fig. 3B). The 27 bp microexon is found within what is the second intron in the more commmon splice form. This intron is not conserved among other AADCs, and is inserted within a region of the BAS-1 protein that is not conserved among AADC proteins. Modeling of BAS-1 protein structure, based on a recent crystal structure of porcine DDC [43], indicates that this region is located at the surface of the protein where it would not interfere with the conserved enzymatic function of the protein (data not shown). We observed that this additional exon is conserved in the *C. briggsae *ortholog of *bas-1 *in genomic sequence (Fig. 3B), although we did not isolate any splice variants with this exon among *C. briggsae bas-1 *cDNAs we sequenced (see also below). We found a single clone that used an alternative splice acceptor 60 bp upstream of the usual splice site for exon 3; this alternate splice introduces a premature stop codon in the coding sequence. This transcript may be a rare, aberrant splice form without functional significance.

### Expression of a *bas-1*::GFP reporter fusion in transgenic worms

**Figure 4:**
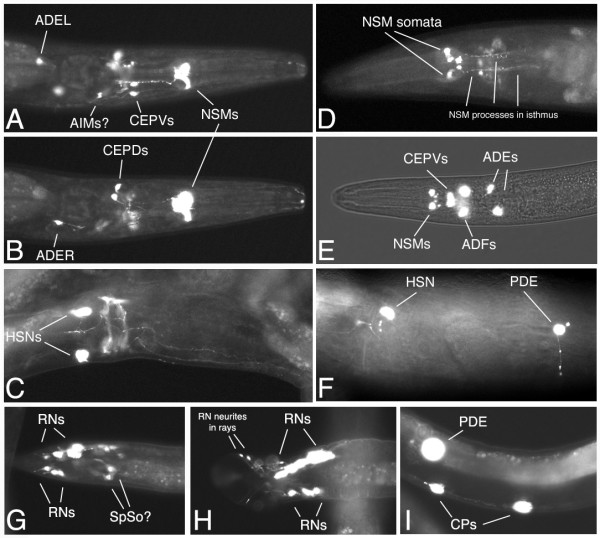
**Expression pattern of a *bas-1*::GFP reporter fusion in transgenic Roller worms. **(Panels A-C are from the same adult hermaphrodite. Ventral is down and anterior to the right.) A. Ventral, slightly oblique view of the head, showing NSMs, CEPDs, ADEL and likely AIMs. B. Same head, higher (more dorsal) focal plane, showing CEPDs and ADER. C. Photomontage showing ventral oblique view of HSNs and their processes in the ventral nerve cord; note also apparent labeling of muscles associated with the vulva. A second worm is immediately adjacent above, obscuring the edge of the worm shown. (Panels D-F: Anterior is to the left.) D. Adult hermaphrodite head, ventral view, chosen to show the characteristic highly varicose processes of the NSM cells within the isthmus of the pharynx. E. Larval head, ventral view with fluorescence and brightfield. This clearly shows the location of the NSM somata in the ventral pharynx, anterior bulb; it also shows the serotonergic ADF neurons not seen in A, B. CEPDs would be seen in a dorsal focal plane in this worm. F. Adult hermaphrodite lateral view of body wall. Ventral is down. Shows HSN and PDE; note PDE process extending ventrally toward the ventral nerve cord and dendrite extending dorsally into postdeirid sensillum. Twisting of the body axis associated with Roller phenotype makes HSN and PDE somata appear at the same lateral level when HSN is actually located sublateral and PDE subdorsal; twisting also takes ventral nerve cord out of plane of focus in the right of the panel. (Panels G – I are from males; anterior is to the right.) G. Late L4 male tail showing ray neurons (RNs) with processes extending into the rays. In some males we saw spicule cell staining likely belonging to spicule socket cells (SpSo). Ventral, slightly oblique view. H. Adult male tail showing RNs and their neurites in rays 7 and 9 on the right side, view ventral, slightly oblique. I. Male-specific ventral nerve cord motoneurons CP5 and CP6, the CP neurons most commonly expressing the transgene. The PDE soma in the lateral body wall is out of the plane of focus.

We examined the pattern of expression of a GFP reporter construct with ~4500 bp upstream of the predicted *bas-1 *translation start site and an in-frame fusion with the 2nd exon, injected with *rol-6(dom) *plasmid into wild-type worms (kindly provided by Ian Hope). Two independent transgenic Roller lines with extrachromosomal arrays had the same pattern of expression. The reporter was reliably expressed in several easily identified cells including the paired serotonergic neurons NSM and HSN and the dopaminergic PDE postdeirid sensory neurons (Fig. 4). NSM processes studded with varicosities were apparent in the isthmus of the pharynx labeled with GFP (Fig. 4A,4D). The egg-laying neuron HSN normally expresses serotonin only in adulthood, and we found the reporter to be expressed in adult hermaphrodites and sometimes late L4 larvae. Often the HSN processes were apparent extending to vulval muscles and anteriorly within the ventral nerve cord (Fig. 4C,4F). We saw a cell we identified as PDE, which is born during L2, only after this stage. In some worms, we saw a PDE process and dendrite, confirming our identification (Fig. 4F).

The *bas-1*::GFP reporter was also expressed in other neurons in the head, around the nerve ring. We believe that all of these cells are known serotonergic and dopaminergic neurons. It was somewhat more difficult, however, to be certain about these identifications since we saw few processes, and even when present we could not always unambiguously associate a process with a particular neuronal soma. Nevertheless, the reporter was expressed in probable dorsal and ventral cephalic sensilla neurons CEPD and CEPV; we sometimes observed as many as four processes extending to the tip of the nose (Fig 4A,4B,4E). We also saw expression in the anterior deirid sensory neurons ADE (Fig. 4A,4B,4E). Less frequently we saw expression in probable ADF and AIM neurons (Fig 4A,4E). We saw as many as 12 neurons (6 bilateral pairs) expressing the reporter in the head of young larvae. This includes all the identified serotonergic (NSM, ADF, AIM) and dopaminergic (CEPD, CEPV, ADE) head neurons excepting the unpaired RIH neuron [10]. In a small number of males examined, we saw expression in male-specific serotonergic and dopaminergic neurons, including up to 6 pairs of ray sensory neurons (RNs) in both adults and late L4 larvae (Fig. 4G,4H). (There are three pairs of serotonergic, and three pairs of dopaminergic RNs among the 18 RNs.) Expression in CP neurons, male-specific ventral cord motoneurons controlling tail curling during mating, was limited and usually weak in the male worms we examined. Six CP neurons are strongly serotonin-immunoreactive in males [16]. At most we saw three posterior cells staining, and usually only one or two posterior cells (CP5, CP6) weakly stained, when expression was present at all (Fig. 4I). We never saw CP staining in L4 animals, and often none even in male worms expressing GFP strongly in the RNs.

### C05D2.4 (*bas-1*) and its downstream homolog C05D2.3

**Figure 5:**
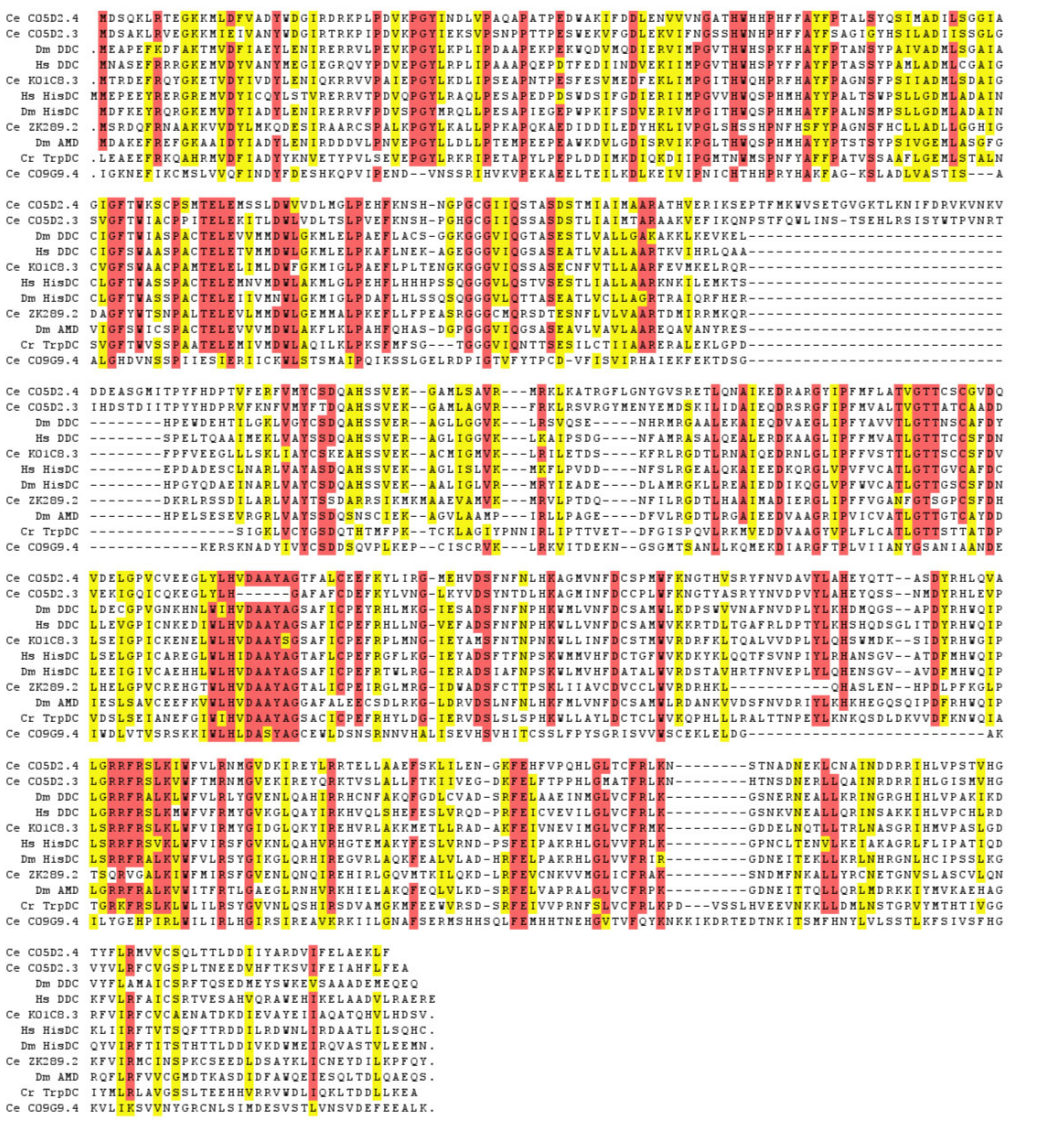
**Alignments of AADC protein sequences with *C. elegans *BAS-1 predicted protein. **Gaps are indicated with a *dash *(-); at the beginning or end of a sequence, *periods *indicate additional sequence upstream or downstream that is not shown. Alignments were performed with CLUSTALW. Abbreviations for species and gene names are the same as listed in the legend for Table 1. For genes with multiple splice forms, the most readily aligned sequence was chosen. Red shading indicates amino acids are identical in ≥ 90% of the aligned sequences. Yellow shading indicates similar amino acids found in that position in ≥ 90% of the aligned sequences.

Just downstream of the *bas-1*/C05D2.4 gene is C05D2.3, the product of an ancient tandem duplication event. The two genes have diverged considerably – being only 59% identical at the amino acid level (Table 1). The genomic structures of the two genes have also diverged. The two genes share four introns, but C05D2.4 has one and C05D2.3 has three introns not found in the other (Fig. 7). Nevertheless, comparisons with other AADC proteins showed that *bas-1*/C05D2.4 is most similar to C05D2.3 and the predicted gene F12A10.3 (Fig. 5, Table 1). The predicted amino acid sequence of C05D2.3 contains one noteworthy gap: it is missing six amino acids from a highly conserved region found in all other PLP-dependent decarboxylases. This sequence, the consensus of which is VDAAYA, contains an aspartate (D) residue that is absolutely essential for function of Rat DDC. Substitution of an alanine or asparagine completely abolishes enzymatic activity, and even the conservative substitution of a glutamate at this site reduces activity to 2% of wildtype [5]. It is therefore unlikely that a C05D2.3 protein could function enzymatically as a typical AADC.

**Table 1:** Pairwise BLAST comparisons with *C. elegans *AADCs.

	C05D2.4	C05D2.3	F12A10.3*	K01C8.3	ZK829.2	C09G9.4	*Ce *GAD
C05D2.4 (*bas-1*)	Score %Id / %Sim	-	-	-	-	-	-
C05D2.3	1674 59 / 75	-	-	-	-	-	-
F12A10.3*	1756 60 / 77	1793 63 / 78	-	-	-	-	-
K01C8.3 (*tdc-1*)	970 37 / 57	844 34 / 54	733 33 / 51	-	-	-	-
ZK829.2	583 29 / 47	541 27 / 46	559 27 / 49	1147 44 / 66	-	-	-
C09G9.4	224 22 / 40	150 18 / 38	190 19 / 40	272 22 / 42	275 23 / 44	-	-
*Ce *GAD (*unc-25*)	210 22 / 38	164 20 / 36	216 23 / 37	299 25 / 43	250 24 / 41	129 20 / 40	-
*Dm *DDC	**1095** **41 / 60**	909 36 / 56	**984** **38 / 58**	1390 50 / 69	883 37 / 57	256 22 / 42	328 24 / 40
*Hs *DDC	1067 41 / 60	**912** **36 / 55**	949 38 / 58	1458 55 / 73	935 39 / 59	233 20 / 42	322 26 / 44
*Dm *HisDC	996 39 / 57	816 33 / 53	807 32 / 54	1348 52 / 70	910 40 / 59	**278** **23 / 42**	339 26 / 44
*Hs *HisDC	988 38 / 56	802 33 / 53	876 34 / 56	1290 48 / 69	920 39 / 59	231 21 / 42	306 26 / 42
*Dm *G30446	971 38 / 57	845 34 / 53	686 30 / 50	**1671** ** 64 / 77**	**1008** ** 40 / 62**	254 20 / 44	n.s.
*Dm *AMD	961 38 / 57	799 35 / 53	668 36 / 53	1124 44 / 63	741 33 / 51	201 20 / 40	278 25 / 41
*Dm *G30445	837 34 / 52	797 35 / 53	814 33 / 53	1407 55 / 71	905 39 / 57	259 22 / 42	n.s.
Cr TrpDC	743 30 / 51	674 28 / 50	735 30 / 52	916 37 / 59	269 31 / 51	272 24 / 43	333 25 / 42
*Dm *GAD	226 22 / 39	207 21 / 38	232 22 / 41	374 27 / 43	226 22 / 41	99 25 / 55	1146 44 / 64
*Hs *GAD67	215 22 / 36	173 19 / 36	192 21 / 36	324 24 / 44	228 24 / 41	86 16 / 41	**1535** **56 / 73**

Comparisons of *bas-1*/C05D2.4 and other pyridoxal-phosphate dependent decarboxylase amino acid sequences were made using "BLAST 2 Sequences" [version 2.2.6,  [73]; Settings (largely default): Matrix – BLOSUM62, Open gap penalty – 11, extension gap penalty – 1, low complexity filtering – OFF). As shown in the table above, on the top line, each comparison shows the blast score; below is the percent identity and percent similarity for the 'alignable' sequence. The highest scoring match (excluding among *C. elegans *AADCs) is indicated in bold. Sequences in the left column are arranged in order of blast score in comparison to C05D2.4 *C. elegans *AADCs are indicated by their predicted gene designation: C05D2.4, C05D2.3, F12A10.3, K01C8.3, ZK829.2 and C09G9.4. Abbreviations: *Ce *– *C. elegans*, *Cr *– *Caranthus roseus *(periwinkle plant), *Dm *– *Drosophila melanogaster*, *Hs *– *Homo sapiens*, DDC – dopa decarboxylase, HisDC – histidine decarboxylase, AMD – Alpha-methyl dopa hypersensitive protein, TrpDC – tryptophan decarboxylase, GAD – glutamate decarboxylase. *An amino acid sequence for F12A10.3 was generated from cDNA sequence by introducing 2 frameshifts to preserve AADC homology in the predicted aa sequence merely for sake of comparison to other AADC's (see text); this sequence is different from predicted sequences in found in Genbank and Wormbase which are based on incorrect cDNA predictions.

**Figure 6:**
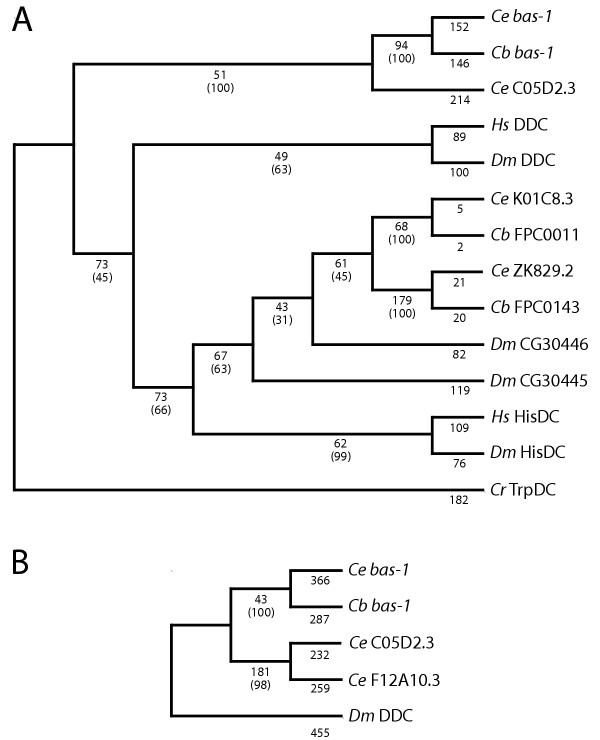
**Phylogenetic trees of AADC protein and nucleotide sequences. **Trees were made from sequences aligned with CLUSTALW. Species and gene names are abbreviated as listed in the legend for Table 1. (A) The single minimum-length tree resulting from a heuristic search using parsimony from alignments of core protein sequences (531 characters) of selected *C. elegans*, *C. briggsae*, Human and *Drosophila *AADCs. *C. roseus *(periwinkle plant) TrpDC was used as an outgroup. Branch lengths are indicated, with bootstrap values using the same search conditions (1000 replicates) in parentheses. The search used the tree-bisection-reconnection (TBR) branch-swapping algorithm; characters were equally weighted. An identical tree topologically was obtained by a branch-and-bound search. *C. elegans *F12A10.3 was excluded from this analysis since it lacks a functional protein sequence (see Fig. 7 and text). Trees determined by distance methods were similar, but rearranged some of branches with low bootstrap values in the tree shown. (B) The single minimum-length tree resulting from a heuristic search using parsimony (same settings as above) of nucleotide sequence alignments (1608 characters) from a subset of AADCs above, with the addition of *C. elegans *F12A10.3. *Dm *DDC was used as an outgroup. Branch lengths and bootstrap values using the same search conditions (1000 replicates) are shown as in A. An identical tree topologically was obtained by a branch-and-bound search.

Because the *bas-1 *and C05D2.3 genes are so close together – only 369 bp from predicted translation stop to predicted translation start – we considered whether they might be expressed as an operon. In *C. elegans *and other nematodes, genes that are very close together (and often functionally related) may be expressed from a single promoter initially as a single primary transcript [44]. Operon transcripts are subsequently processed to yield separate mRNAs. The first gene in an operon is trans-spliced to the leader sequence SL1; downstream genes are typically spliced to a slightly different leader sequence termed SL2. We would expect to find C05D2.3 transcripts trans-spliced to SL2 if it is a downstream gene in an operon with *bas-1*. We were unable to isolate either SL1 or SL2-spliced transcripts from C05D2.3 by RT-PCR, although we did isolate a partial cDNA using internal primers. DNA microarray experiments suggest the gene is not expressed above background levels, unlike C05D2.4/*bas-1 *(Table 2). Furthermore, a global analysis of expression specifically designed to identify operons did not select C05D2.4 and C05D2.3 as likely members of an operon [45]. Since genes comprising an operon should be expressed at similar levels, these data provide no support for the idea that *bas-1 *and C05D2.3 constitute an operon.

**Table 2:** *C. elegans *AADC genes expression and *C. briggsae *orthologs.

***C. elegans *AADC**	***C.e. *cDNAs**	***C.e. *Microarray**	***C. briggsae *ortholog**	***C.b. *cDNAs**
C05D2.4 (*bas-1*)	+(3) ^a,b,c,d^	+	FPC2187 (84,978 / - strand)	+ ^a^
C05D2.3	+ ^a,c^	-	none	NA
C09G9.4	+ ^d^	-	FPC4079 (~28,330 / + strand)	-
F12A10.3	+(2) ^b,d^	-	none	NA
K01C8.3 (*tdc-1*)	+(2) ^c,d^	+	FPC0011 (663,153 / + strand)	+
Y37D8A.23 (*unc-25*)	+(3) ^c,d^	+	FPC4030 (765,572 / - strand)	-
ZK289.2	+ ^c,d^	-	FPC0143 (1,747,392 / - strand)	-

*C. e. *cDNAs: Parentheses indicate number of splice forms found. ^a^cDNAs found via our RT-PCR experiments and ^b^our sequencing of ORFeome project clones. ^c^*C. elegans *EST project. ^d^Worm ORFeome project. Microarray: Expression levels at all developmental stages as shown by *C. elegans *microarray experiments found in Wormbase. "+" indicates significant expression at some stage; "-" indicates no expression above background detected at any stage. *C. briggsae *AADC orthologs: We did TBLASTN searches of the *C. briggsae *whole genome shotgun assembly (cb25.agp8) on the Sanger Centre *C. briggsae *blast server using complete predicted amino acid sequences for each *C. elegans *AADC gene. *C. briggsae *genes are designated by contig location, first nucleotide of predicted coding sequence, and strand, based on predicted *C. elegans *sequence. In each case, alignments showed extended regions of 100% or near 100% amino acid identity beginning at the site indicated. (We did not locate the beginning of the *C.b. *C09G9.4 ortholog; alignments did not identify a matching site for the first 11 *C. elegans *amino acids). For C05D2.3 and F12A10.3, the best matches in *C. briggsae *were the C05D2.4 ortholog (FPC2187), followed by the K01C8.3 ortholog (FPC0011); these two genes appear to be absent from *C. briggsae*. We have isolated an SL1-spliced *C. briggsae bas-1 *cDNA by RT-PCR (S. DePaul & C. Loer, unpublished); *C. briggsae tdc-1 *ESTs are in GenBank. NA – not applicable.

### The *bas-1*-AADC and other AADC genes in *C. elegans*

We compared the predicted amino acid sequences of five other *C. elegans *AADC-like genes revealed by deletion mapping [46] and by whole genomic sequencing [24], along with a previously identified *C. elegans *glutamate decarboxylase (GAD) gene, *unc-25 *[47] to related PLP-dependent decarboxylases from other organisms. Some of the *C. elegans *genes are clearly closely related to other AADCs, whereas others are more divergent (Fig 5, Table 1). All contain the core conserved domain (PFAM 00282) defining this group of PLP-dependent decarboxylases. None of the AADC or GAD predicted proteins in *C. elegans *appears to have a signal sequence.

The protein predicted from K01C8.3 is now believed to encode a tyrosine decarboxylase (*tdc-1*) used for tyramine and octopamine synthesis, which both appear to be used as neurotransmitters in *C. elegans *[48,49]. The best match to K01C8.3/*tdc-1 *is a predicted *Drosophila *AADC-homologous protein of unknown function (G30446). Interestingly, K01C8.3/*tdc-1 *shows a stronger match to known DDCs than any of the other *C. elegans *AADCs, including C05D2.4 (Table 1), although it is equally similar to known histidine decarboxylases (HisDCs). The strongest match of C05D2.4/*bas-1 *(outside of nematodes) is to insect and mammalian DDCs, but again the match is only slightly better than to HisDCs. The predicted genes C05D2.3, F12A10.3 and ZK829.2 also have about the same level of identity and similarity to known AADCs and HisDCs. The ZK829.2 predicted protein, however, is much larger (830 AA) than a typical AADC, having extended N- and C-terminal domains not found in other PLP-dependent DCs. Most of ZK829.2 predicted coding sequence is confirmed by cDNA sequences, suggesting that the predicted protein 'extensions' likely are real.

The predicted gene C09G9.4 is the most divergent from known AADC's with only 20 – 24% amino acid identity; it is even more divergent than *C.e. *GAD/*unc-25*. It also appears to lack the absolutely conserved Lys of PLP-dependent decarboxylases, although it otherwise retains considerable homology with the conserved domain of this family of proteins. There are no similar proteins among other organisms to provide clues about a possible function for this gene; C09G9.4 is a truly novel member of the group II PLP-dependent enzyme family. Proteins with a similar level of divergence with AADC (~20% identity over a few hundred amino acids) include other group II PLP-dependent enzymes such as sphingosine-1-phosphate lyase and cysteine sulfinic acid decarboxylase. C09G9.4, however, has very little or no significant similarity to these other enzymes. The *C. elegans *GAD/*unc-25 *predicted protein has a strong match to identified *Drosophila *and mammalian GADs (Table 1), and is found as a single copy. There are no other GAD-like genes in *C. elegans *such as cysteine sulfinate decarboxylase, which is the rate-limiting enzyme in taurine synthesis, and the closest non-AADC relative to GAD in the vertebrates [50].

### Comparison of *C. elegans *and *C. briggsae *AADC genes

We performed BLAST searches of a *C. briggsae *whole genome shotgun assembly using predicted protein sequences of all six *C. elegans *AADC genes and the *unc-25*/GAD gene. We found five orthologous genes in *C. briggsae *– four AADC homologs and one GAD homolog (Table 2). All of these matches included 100% or near 100% identity over extended regions of aligned predicted amino acid sequences, and were paired with high confidence in phylogenies (Fig. 6). Using a core AADC sequence for alignments and tree building, we found that the *bas-1 *orthologs have evolved more quickly than some of the other AADC's. The *C. elegans *gene K01C8.3 and its ortholog, for example, are 98% identical in this core region (vs. 91% identity for *bas-1 *orthologs). Most of the divergence between K01C8.3 and its ortholog is in N- and C-terminal extensions that are not found in other AADC's. The *C. elegans *C09G9.4 and *C. briggsae *ortholog are even less similar to one another than are the *bas-1 *orthologs.

**Figure 7:**
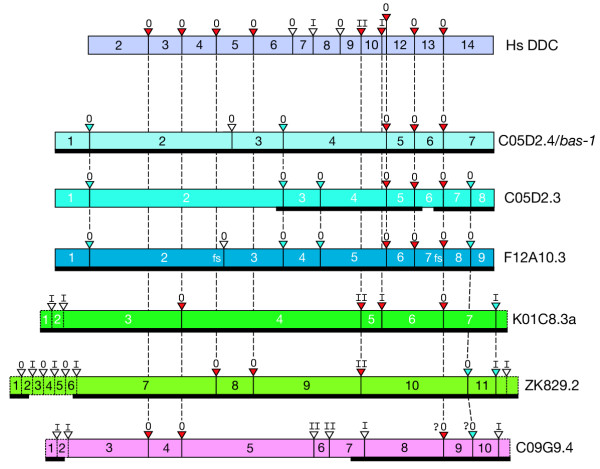
**Genomic structure of *C. elegans *AADC genes compared with Human DDC. **Rectangular blocks represent coding exons of the genes indicated (relative size of exons is approximate). Red triangles indicate ancient conserved introns found in both Human DDC and at least one of the *C. elegans *AADC genes; blue triangles indicate introns conserved among *C. elegans *AADC genes; and open triangles indicate non-conserved splice sites (comparing only among the genes shown). Roman numerals above triangles indicate the phase of the intron. Vertical dashed lines between solid triangles indicate splice sites conserved between at least two genes. Diagonal dashed lines indicate probable conserved sites that are shifted by 2–3 amino acids relative to the other splice site. Alignments of homologous splice sites are based on amino acid multiple alignments of the predicted proteins; insertions and deletions are ignored in the drawing. No alternative splicing is indicated; the most readily "alignable" version of each gene was used in cases with multiple splice variants. Dashed boxes at the ends of genes indicate non-AADC homologous extensions unique to the given gene. The most divergent AADC, C09G9.4, is more difficult to align; assignments of splice sites on either side of exon 9 as conserved are more tentative (indicated by question marks). In a few cases where gaps occur in the protein sequence alignments at intron-exon boundaries, introns marked as homologous only begin or end at an homologous location. The splicing pattern shown is fully supported by cDNA sequences for C05D2.4/*bas-1*, F12A10.3, K01C8.3, ZK829.2 ; the pattern is supported by partial cDNA sequences for C05D2.3 and C09G9.4. The extent of supporting cDNA sequence is shown by the heavy black line beneath the colored blocks. F12A10.3 is a special case in that frameshifts (indicated by 'fs') occur in the cDNA relative to the AADC homologous sequence. The first frameshift occurs at a site where many AADCs are spliced (and where a splice is incorrectly predicted by gene prediction programs), and the second at a splice junction.

Our most striking observation is that *C. briggsae *appears to lack orthologs for the *C. elegans *predicted genes C05D2.3 and F12A10.3. This suggests that gene duplications giving rise to these two genes, which are most closely related to *bas-1*/C05D2.4, occurred either in the *C. elegans *lineage after its split with the *C. briggsae *lineage, or that *C. briggsae *lost both C05D2.3 and F12A10.3 orthologs (or their common ancestor) following the split. Using phylogenetic analysis of aligned amino acid and nucleotide sequences, we found that C05D2.3 and F12A10.3 share a common ancestor and that the gene duplication giving rise to *bas-1 *and C05D2.3/F12A10.3 likely occurred prior to the *C. elegans/C. briggsae *divergence. This is also suggested by the pattern of introns in the genes. [We have confirmed the splicing pattern of *C. briggsae bas-1 *by isolating a cDNA (S. DePaul & C. Loer, unpublished data).] The *C. elegans *and *C. briggsae bas-1 *genes have identical genomic structure which differs from that of C05D2.3 and F12A10.3, which are more similar to one another (Fig. 7). Therefore C05D2.3 and F12A10.3 (or their common ancestor) were retained in the line leading to *C. elegans *but lost in the *C. briggsae *line. The original duplication event giving rise to the tandem copies of C05D2.4 and C05D2.3 on chromosome III probably occurred via an unequal crossing-over or similar event. The duplication creating F12A10.3, which is found on chromosome II, presumably occurred subsequently. We noted no homology of other predicted genes downstream of F12A10.3 and C05D2.3 that might suggest an event duplicating more than the AADC gene.

The retention of the genes and their expression in *C. elegans *suggests that they may have acquired a new function that is under selection, retain a subfunction of the AADC, or instead that they are still in the process of being lost. After sequencing F12A10.3 cDNAs (courtesy of the ORFeome Project), we found that current splicing predictions for the gene were incorrect. We sequenced six F12A10.3 clones and found two slightly different splicing patterns, both different from Genefinder and Intronerator predictions. The two types of clones differed only in whether a final intron was removed or not. We found 4 clones with 9 exons, and 2 clones with 8 exons. The failure of the gene prediction programs in this case is likely to be due to their preference for creating functional transcripts. All F12A10.3 cDNAs instead appear to be non-functional: they have frameshifts relative to AADC-homologous reading frames. The first frameshift occurs in the second exon and quickly leads to a premature stop codon. At best F12A10.3 transcripts would result in a 158 amino acid protein that could not function as an AADC. F12A10.3 therefore appears to be an expressed pseudogene. DNA microarray experiments and representation in cDNA sequencing projects suggest that F12A10.3, like C05D2.3, is likely expressed at a low level (Table 2).

In order to assess whether the *bas-1*-like genes C05D2.3 and F12A10.3 might be under reduced selection pressure, we calculated the ratio of non-synonymous to synonymous substitutions (K_A_/K_S_) comparing the *bas-1 *orthologs and *bas-1*-like genes. We also calculated these values for the other AADC ortholog pairs from *C. elegans *and *C. briggsae *(Table 3). K_A_/K_S _< 1 indicates purifying (negative) selection, K_A_/K_S _= 1 indicates no selection (as in true pseudogenes), and K_A_/K_S _< 1 indicates Darwinian (positive) selection. K_A_/K_S _for 8179 *C. elegans *and *C. briggsae *ortholog pairs had a mean value of 0.06, indicating most genes are under purifying selection [37]. We found that the *bas-1 *genes are under purifying selection (K_A_/K_S _= 0.039), but the *bas-1*-like genes appear to be under reduced selective pressure; the average K_A_/K_S _for comparisons with *bas-1*-like genes was 0.148, more than three times the value of the *bas-1 *ortholog comparison. The proportion of observed to potential non-synonymous substitutions (pN) among the *bas-1*-like gene comparisons was similarly much higher than for the *bas-1 *orthologs.

**Table 3:** Synonymous vs. non-synonymous codon substitution between *C. elegans *and *C. briggsae *AADC orthologs and *bas-1*-like paralogs.

***Ce*, *Cb *AADCs compared**	**Codons**	**pS**	**pN**	**K_A_/K_S_**
*bas-1*	521	0.68	0.07	0.039
C05D2.3, F12A10.3, bas-1*	521	0.71 ± 0.03	0.26 ± 0.02	0.148 ± 0.044
ZK289.2	833	0.71	0.09	0.043
C09G9.4	507	0.74	0.12	0.037
*tdc-1 *full length	626	0.79	0.03	NA
*tdc-1 *core	474	0.82	0.02	NA
*tdc-1 *N, C terminals	152	0.71	0.07	0.035

Nucleotide alignments of *C. elegans *and *C. briggsae *genes were analyzed by SNAP software (see Methods). Only the *C. elegans *member of the ortholog pair is named. pS = proportion of observed/potential synonymous substitutions; pN = proportion of observed/potential nonsynonymous substitutions. NA – not applicable (cannot be calculated when pS > 0.75). *Includes all pairwise comparisons (n = 5) except *C.e. *vs. *C.b. bas-1*. Values are mean ± SD (strict statistical comparison with other values is not intended, as K_A_/K_S _values are not distributed normally).

Two other AADC ortholog pairs showed strong purifying selection at work, with values like that calculated for the *bas-1 *orthologs (Table 3), but a value could not readily be calculated for the *tdc-1 *orthologs. In all the AADC ortholog comparisons, the proportion of observed to potential synonymous substitutions (pS) was near mutational saturation (pS > 0.75); K_A_/K_S _cannot be calculated when pS > 0.75. Thus, a value could not be calculated either for full-length *tdc-1 *alignments, or using a *tdc-1 *core sequence. We were able to calculate a value by aligning the N- and C-terminals sequence of the *tdc-1 *orthologs (Table 3). These regions of the protein are under levels of selection like the other AADCs, whereas the core has a very low rate of non-synonymous substitution, consistent with the high level of amino acid conservation in this region of the protein.

## Discussion

Our experiments demonstrate that the predicted gene C05D2.4, which encodes an aromatic L-amino acid decarboxylase (AADC), corresponds to the genetically-defined *bas-1 *gene. Serotonin immunoreactivity is restored in *bas-1 *mutants by DNA containing an intact C05D2.4 gene, but not with DNA mutated in C05D2.4. The adjacent AADC-homologous gene, C05D2.3, is not needed to rescue *bas-1 *mutants. The *bas-1 *gene is therefore likely to encode the serotonin- and dopamine-synthetic AADC of *C. elegans*. Although we did not test for rescue of dopamine expression, it is likely that *bas-1 *encodes the same AADC required for DA synthesis. Mutants with point mutations in C05D2.4 – *bas-1 *alleles *n2948 *and *n3008 *– have been shown previously to be DA-deficient [17], and neither of these appears to contain mutations in the C05D2.3 gene. Furthermore, AADC proteins from other animals have been consistently shown to catalyze both 5HTP and L-dopa decarboxylation reactions [3]. Finally, a *bas-1 *reporter construct is expressed both in identified serotonergic and dopaminergic cells.

The *bas-1 *gene is expressed in at least two alternatively spliced forms, one of which appears to be less common and contains a small additional 27 nucleotide exon. The short segment of protein encoded by the additional exon, and the surrounding region are not found in other AADC proteins, suggesting a novel function for this region of the AADC protein. In other organisms, the serotonin- and dopamine-synthetic AADC genes have alternative splicing that result in tissue-specific protein isoforms. Currently we have no indication that *bas-1 *is expressed in any cells other than serotonergic and dopaminergic neurons, and no information about the functional significance of this alternative splicing.

AADC has received somewhat less attention with respect to the regulation of serotonin and dopamine synthesis than the specific, rate-limiting synthetic enzymes tryptophan hydroxylase and tyrosine hydroxylase [51]. This is in part due to the view that AADC activity is not limiting, and that its activity is not regulated. Regulation of AADC activity by protein kinase A-dependent phosphorylation has recently been proposed based on *in vitro *experiments [52], although its functional significance has been questioned [53]. Our examination of the predicted BAS-1 protein revealed several potential phosphorylation sites that are highly conserved, although none fit the consensus sequence for PKA phosphorylation. Any possible regulation of *C. elegans *AADCs by phosphorylation remains speculation.

### Possible functions of other AADC homologous genes in C. elegans

We compared the protein sequences of other predicted AADCs in *C. elegans *with those of other organisms in order to guess about their possible functions. This is particularly relevant because all *bas-1 *mutants retain weak, residual serotonin immunoreactivity ([13]; C. Loer, unpublished) suggesting that other enzymes may be able to carry out the same reaction. This would not be surprising since animal AADCs tend to have broad specificity [3]. Based purely on sequence homology, it seems that predicted genes K01C8.3 and ZK829.2 could act as AADCs or as HisDCs. In fact, the predicted gene K01C8.3 is now believed to be a tyrosine decarboxylase and has been named *tdc-1 *[48]. If correct, then its best match in *Drosophila *(G30446), an uncharacterized AADC homolog, is likely to encode the fly's octopamine-synthetic tyrosine decarboxylase. It has long been known that a separate gene encoded this enzymatic activity in flies, since the activity is still detectable in *Ddc *deletion mutants [4]. It will be interesting to see whether such tyrosine decarboxylases in animals have more restricted substrate specificity, such as the tyrosine and tryptophan decarboxylases in plants [54], or are more similar to typical animal AADCs with a broad specificity. Tighter substrate specificity of a *tdc-1 *protein could be reflected in the much slower rate of amino acid substitution seen in its *C. elegans *&* briggsae *orthologs than in the *bas-1 *orthologs which encode more 'promiscuous' enzymes.

Whether *C. elegans *or other nematodes make the neurotransmitter histamine, and therefore need a HisDC enzyme, is unclear. Although histamine has been reportedly isolated from *C. elegans *[55], this observation is unique among nematodes, and has not subsequently been confirmed. There is no particularly good candidate for a HisDC in *C. elegans*. The ZK829.2 predicted protein may be most closely related to *tdc-1 *in its core sequence, although its long N- and C-terminal extensions are perhaps suggestive of a new function. Unfortunately, transgenics with reporter fusions of this gene to date have shown no expression, the pattern of which might suggest a function (C. Loer, unpublished; M. Alkema, personal communication). As with *tdc-1*, *C. elegans *ZK829.2 and its *C. briggsae *ortholog have also evolved more slowly than the *bas-1 *orthologs. A recent analysis of eukaryotic AADC sequences that includes the *C. elegans *ZK829.2 and its *C. briggsae *ortholog as the only nematode representatives clearly demonstrates that AADC genes can evolve at very different rates, and that a constant "molecular clock" cannot be assumed in phylogenetic analyses [56].

Finally, since the C09G9.4 predicted protein is so highly divergent from the typical AADC, and lacks a critical lysine residue that binds the PLP cofactor, it is unlikely to be an AADC enzyme. It has a similar level of divergence from genuine AADCs as do other group II PLP-dependent enzymes such as cysteine sulfinic acid decarboxylase, to which it has little or no similarity. Whatever the function of a C09G9.4-encoded protein, it appears to represent a new PLP-DC-related protein; sequencing of more genomes may yet reveal additional members.

### Duplicate gene retention and loss in *Caenorhabditis*

We found that the closest relatives of C05D2.4/*bas-1 *in *C. elegans*, the genes C05D2.3 and F12A10.3, are missing from *C. briggsae*. Furthermore, phylogenetic analysis indicates the two extra genes did not arise in the *C. elegans *line, but were present (or their commmon ancestor was present) in the species that gave rise to both the *C. elegans *and *C. briggsae *lines. Finally, careful examination of the cDNAs and predicted protein sequences of C05D2.3 and F12A10.3 reveals that neither is likely to be functional as an AADC: the former lacks critical amino acids and the latter can encode only a truncated protein. Both are expressed, based on the presence of cDNAs, but probably at a very low level, which is not above background in microarray experiments. It is possible that the duplicate genes are functionally 'lost' in *C. elegans *as well.

The features of C05D2.3 and F12A10.3 raise a number of interesting questions about the fate of duplicate genes, and the true nature of many 'predicted genes' in *C. elegans*. Taking a random sampling of predicted genes and generating transgenics with reporter fusion constructs (in order to determine a pattern of expression), Mounsey and colleagues [57] found that a much higher percentage of recently duplicated genes than conserved or unique genes failed to show expression. Assuming that failure of expression was no more likely among recently duplicated genes for technical reasons, this meant that many more of these are in reality not expressed. The numbers suggested that up to 20% of annotated, predicted genes in *C. elegans *may be pseudogenes. In fact, careful inspection of recently duplicated genes showed that many were actually pseudogenes, like we found to be the case for F12A10.3. Overall, close inspection of predicted genes revealed at least 4% were pseudogenes.

So, why are C05D2.3 and F12A10.3 still present in *C. elegans *if they lack a function? *C. briggsae *and *C. elegans *may have diverged 80 – 110 million years ago [37,38]. Since the *bas-1*-like gene or genes were likely present in the common ancestor of *C. elegans *and *C. briggsae*, then there seems to have been ample time for loss in the *C. elegans *line. Under a simple model of gene loss following duplication, only a few million generations would be the mean time to fix a null allele of the gene duplicate [58]. In *Caenorhabditis*, a million generations could be completed in 10,000 years or less. This seems to suggest that the downstream duplicate of *bas-1 *(ancestor of C05D2.3) may have continued to function for a considerable time after the duplication, perhaps by gene conversion which might have continued until sufficient divergence from *bas-1 *[59]. Loss of the critical six amino acids occurred after the second duplication giving rise to the ancestor of F12A10.3, since the appropriate sequence is still present there (although frame-shifted). It is also possible that the C05D2.3 gene retains some function. The gene still encodes a respectable protein, albeit one that seems unable to function as an AADC. It has diverged considerably from *bas-1*, but has not accumulated stop codons and frameshifts expected for a pseudogene. Walsh [60] has proposed that fixation of an allele with an advantageous new function, vs. becoming a pseudogene, may be the fate of many duplicate genes even when such mutations are rare, given a population that is sufficiently large.

C05D2.3 and F12A10. 3 seem to have been retained longer than expected. Lynch and Force [61] proposed that the unexpectedly high rate of gene duplicate retention in eukaryotic genomes is due to 'subfunctionalization' – the retention of a portion of the original single gene's function by each of the duplicates, which then complement one another. Although this was suggested to occur primarily by regulatory mutations that partition expression of the genes spatially, other forms of subfunctionalization could also occur. Another possible reason for retaining such genes is the presence of non-coding regulatory functions associated with transcription and splicing of these sub-functional transcripts that affect the transcription of other nearby genes, although a *bas-1*::GFP construct is expressed well without such sequences *in cis*.

Our analysis of synonymous vs. non-synonymous substitutions indicates that the *bas-1*-like genes C05D2.3 and F12A10.3 are under relaxed selection relative to *bas-1 *and other AADCs. It should be noted that precise quantitative comparisons cannot be made with the results presented in the *C. briggsae *whole genome analysis [37], since we used a different method of calculating K_A_/K_S_; however our calculations indicate that *bas-1 *and the other AADC's, like most genes in the *Caenorhabditis *genomes, are under strong purifying selection. Even if both C05D2.3 and F12A10.3 are now pseudogenes, some significant period of time during which they functioned and were under purifying selection could act to obscure this fact in an analysis of K_A_/K_S_. Even if C05D2.3 has acquired a new, adaptive function, such a new function might result from changes in only a few sites in the protein, and so again this could be obscured by a majority of sites under purifying selection. With the sequencing of three related *Caenorhabditis *species it will be interesting to learn of the fates of *bas-1 *and the *bas-1*-like genes in other lines.

## Conclusions

The *bas-1 *gene encodes a serotonin- and dopamine-synthetic AADC enzyme in *C. elegans*. The *C. elegans *genome possesses five other AADC-homologous genes, two of which are closely related to *bas-1*. These *bas-1*-like genes are missing, however, from the congeneric *C. briggsae*, and evidence suggests that, despite their persistence in *C. elegans*, the genes do not encode functional AADC proteins. Since one or more of the *bas-1*-like genes was likely present in the common ancestor of *C. elegans *and *C. briggsae- *which may have diverged over 80 million years ago – it is unclear why the *bas-1*-like genes have been retained in the *C. elegans *line. This is another example of unexpected retention of duplicate genes in eukaryotic genomes.

## Methods

Routine culturing of *Caenorhabditis elegans *was performed as described by Brenner [62]. Nomenclature used here for *C. elegans *genetics conforms to the conventions set forth by Horvitz *et al*. [63]. Strains used include N2 (wild type); CB1490: *him-5(e1490)V*; MT7988: *bas-1(ad446)III*; MT7990: *bas-1(n2948)III*; MT8002: *bas-1(n3008)III*; LC7: *bas-1(pa4)III*; LC33: *bas-1(tm351)III*. The *him-5(e1490) *strain generates approximately 30% males by increased X chromosome non-disjunction [64], but is otherwise essentially wild-type.

### Mutant allele sequencing

Mutations were identified by PCR-amplifying small regions from genomic DNA, then sequencing purified PCR product. We isolated genomic DNA (Puregene Kit, Gentra Systems) from four *bas-1 *mutant strains (alleles *ad446, n2948, n3008, pa4*) and then PCR-amplified small segments of C05D2.4 and C05D2.3 protein-coding sequence. Five pairs of primers were used to survey the C05D2.4 gene (A+B, E+F, G+H, P+Q, R+S; see below for primer sequences) and 4 pairs for C05D2.3 (C+D, K+L, M+N, P+Q). Bands of the predicted size were excised from 2% agarose gels and purified with GeneClean (Bio101/Qbiogene), then sequenced. All mutations were confirmed by sequencing both strands for two independent PCR reactions. All PCR and sequencing primers were designed using the program Primer3 (; [65]). Primer sequences were as follows. Within C05D2.4: C05D2-A: gaggaaactcaaggcgacac; C05D2-B: tgttgatggaaccaagtgga; C05D2-E: cgtccttttctctttgcgac; C05D2-F: tggctccgacttgattctct ; C05D2-G: ttacaattaggccgcaaacc; C05D2-H: ccacctgaactgtggtgatg; C05D2-P: ggactcacatgtttccgattg; C05D2-R: ttagacgttggttgcacgag; C05D2-S: attggcgagcagtcaaagtt ; C05D2-T: tcttatgggattaccagaac; C05D2-U: ctacataaagctggaatggt; C05D2-V: gtttcctaaaaatccacgtg; C05D2-W: atgatcgattgatagctgag. Within C05D2.3: C05D2-C: ctaggtgcctttgccttctg; C05D2-D: caagagacgctcgttgtcag; C05D2-K: gccatctaatcctccaacca; C05D2-L: acattgctcccttttcaacg [note that primer L can also prime within C05D2.4]; C05D2-M: ccatcaactttccaatggct; C05D2-N: tctcgacgcccatatttctc; C05D2-Q: ccaattccagcggagaagta.

### Microinjection to create transgenics

All DNAs for microinjection were purified with Qiagen tip20 columns. Experimental DNAs were co-injected with the dominant marker plasmid pRF4 containing the mutant gene *rol-6(su1006) *[66] into N2 (wildtype), CB1490, or *bas-1 *mutant worms. Progeny of injectees that express the *rol-6 *dominant plasmid have the easy-to-identify Roller phenotype which results in worms with a helically-twisted body along the anterior-posterior axis. Roller transgenic worms typically carry co-injected DNAs. Transgenic Roller progeny were isolated and propagated; rescue of *bas-1 *mutants was scored by staining with serotonin antiserum as described previously[16,67].

### Mutant rescue with subclones of cosmid C05D2 and plasmid C05D2XN; GFP Reporter Construct

The rescuing plasmid C05D2XN contains a 15.8 kbp genomic DNA insert (XhoI to NheI) derived from the cosmid C05D2, and contains both C05D2.4 and C05D2.3 predicted genes. A number of deletions of C05D2XN were made to test for rescue of *bas-1 *mutants. Clones were analyzed by restriction digests. We also made C05D2XN derivatives in which either C05D2.4 and C05D2.3 was mutated to introduce a premature stop in coding sequence. Clones were sequenced to determine the nature of the introduced mutation. Clone pCL6991 had a 4 bp deletion in the second exon of C05D2.4; pCL7991 had a 2 bp insertion in the first exon of C05D2.3; each causing a frameshift mutation. In the clone pCL7003, derived from the rescuing plasmid pCL3001, almost the entire C05D2.3 coding sequence is deleted. The *bas-1 *GFP reporter construct (within transgenics) was kindly provided by Ian Hope, and consists of a PCR-generated fragment of C05D2 with 4595 bp upstream of the predicted translation start site and 403 bp protein coding region to make an in-frame protein fusion in the 2nd exon. The construct was created by a multi-site recombination reaction with the C05D2 fragment, PCR-generated GFP, and vector using the Invitrogen Gateway cloning system (I. Hope, personal communication) as continuation of a project to determine expression patterns for *C. elegans *genes through reporter gene technology [57].

### RT-PCR and cDNA clones

We isolated RNA for RT-PCR from mixed stage CB1490 worms. (The CB1490 strain has ~30% males, whereas wildtype N2 has ~0.2% males. Since adult males have 13 more serotonergic neurons and 6 more dopaminergic neurons than hermaphrodites, we reasoned that these worms might express more *bas-1 *mRNA.) Worms were isolated from six 100 mm NGM plates, washed several times with M9 buffer, and pooled to form a ~100 μl pellet of worms. The pellet was mixed with 175 μl RNA lysis buffer (SV Total RNA Isolation System, Promega), frozen and ground to a fine powder with a pestle and mortar cooled with liquid nitrogen. The powder was recovered in a fresh microfuge tube, mixed with 350 μl SV RNA dilution buffer and centrifuged to remove debris. The cleared lysate was transferred to a new tube and precipitated with 200 μl 95% ethanol and applied to the SV spin column assembly. The remaining RNA purification was performed exactly as described for the SV System "RNA Purification by Centrifugation." Purified RNA was eluted from the spin column with 100 μl nuclease-free water. RNA was converted to cDNA in a 80 μl reaction containing 800 Units of M-MLV Reverse Transcriptase (Life Technologies/BRL), 16 μl 5X RT buffer, 25 mM dNTPs, 80 Units RNAsin (Promega) 2.0 μg random hexamer primers (Life Tech), and 50 μl purified RNA (from above). *Bas-1*(C05D2.4) and C05D2.3 cDNAs were amplified by PCR from *him-5 *cDNA produced as described above, typically using Failsafe PCR mixes (Epicentre Technologies). Conditions for PCR were 94°C (1 min.), 50°C (1 min.), 72°C (3 min.) for 40 cycles, then 72°C (10 min.). Primers used to amplify C05D2.4 cDNAs were SL1-B + C05D2-L and SL1-B + C05D2-B; a partial C05D2.3 cDNA was amplified with primers C05D2-M and -N. Spliced leader primer sequences were as follows: SL1-B: AAAGGATCCTTTAATTACCCAAGTTTGAG; SL2-B: AAAGGATCCTTTTAACCCAGTTACTCAAG. Appropriately-sized bands were isolated with GeneClean or Ultrafree-DA (Millipore), then cloned directly into the pCRII-TOPO vector using the TOPO-TA Cloning kit (Invitrogen). From seven of our RT-PCR derived clones we sequenced, we found two different splice variants different form the Genefinder prediction (see results).

We also obtained cDNA clones from the ORFeome project [40] and the *C. elegans *EST project (courtesy Yuki Kohara). DNA we received from the ORFeome project is purified from a pool of transformants derived from ligation and transformation of their original RT-PCR, thereby allowing the isolation of internal splice variants from the mix. We transformed with this DNA and isolated several clones. We sequenced two ORFeome clones completely; six more clones were partially sequenced. Interestingly, each of the 8 clones appeared to have at least one mutation (compared to known genomic sequence). Since we found one splice variant containing the 27 bp microexon from among the eight clones that we sequenced, we analyzed 29 additional ORFeome project-derived clones by PCR from single isolated bacterial colonies. Amplification of the region between primers C05D2-T and -L allowed us to distinguish between clones with or without the 27 bp microexon based on product size (464 bp versus 437 bp). Seven of 29 clones were the larger size, so likely contained the 27 bp microexon. None of the ORFeome clones analyzed by sequence or PCR appeared to use the alternative exon 3 splice acceptor noted above. Finally, the two 'YK' cDNA clones from the *C. elegans *EST project that we examined are from a 'full-length' capped library, and each has an SL1 leader and a poly-A tail. We found that each *bas-1 *'YK' clone, however, contained a different internal deletion (overall abnormality of these clones is reported at ~5% – J. Theirry-Meig, personal communication). Each of the deletions was adjacent to a short repeated sequence.

### Sequence Analyses

*C. elegans *genomic and predicted cDNA sequences were retrieved from ACeDB, WormBase, and/or GenBank. Blast searches and Blast2 comparisons were performed using the NCBI Blast server. *C. briggsae *genomic sequence was searched using TBLASTN of the 7/12/02 shotgun assembly . Assembly and consensus sequence determination of our own cDNA sequences was done using the program SeqMan (DNAStar, Madison, WI). Some information about ORFeome clones was retrieved from WorfDB (; [68]). Phosphorylation predictions were made with NetPhos 2.0  and PhosphoBase . Signal sequence predictions were performed with SignalP v.1.1 (, [69]). Multiple sequence alignments were performed primarily with CLUSTALW , with some manual adjustments. Phylogenetic analyses were performed with PAUP* version 4.0b10 (Sinauer Associates, [70]). Some alignments of cDNAs with genomic DNA to determine intron locations were performed with SIM4 [71]. Estimates of rates of synonymous and non-synonymous substitutions were made with SNAP (Synonymous/Non-synonymous Analysis Program –  using pairwise or multiple sequence nucleotide alignments generated with CLUSTALW and adjusted manually. This program uses the method of Nei and Gojobori [72].

## List of Abbreviations

AADC – aromatic amino acid decarboxylase

DDC – dopa decarboxylase

HisDC – histidine decarboxylase

GAD – glutamic acid decarboxylase

TrpDC – tryptophan decarboxylase

PLP – pyridoxal 5'-phosphate

NSM – neurosecretory motoneuron

HSN – hermaphrodite-specific neuron

PDE – postdeirid sensory neuron

ADE – anterior deirid sensory neuron

RN – ray sensory neuron

CEPD, CEPV – dorsal or ventral cephalic sensory neuron

ADF – amphid sensory neuron, dual cilia, designation F

AIM – ring interneuron, designation M

RIH – ring interneuron (unpaired), designation H

CP – posterior daughter of designation C cell, male-specific ventral cord motoneuron

## Authors' contributions

EH prepared deletion and expression constructs, cloned and sequenced bas-1 cDNAs and mutant alleles, participated in anti-serotonin staining and genetics, and drafted the manuscript. CL conceived and directed the study, generated transgenics by microinjection, performed sequence and phylogenetic analyses, and completed the manuscript. Both authors read and approved the final manuscript.
